# The use of telemedicine in the PICU: A systematic review and meta-analysis

**DOI:** 10.1371/journal.pone.0252409

**Published:** 2021-05-28

**Authors:** Maria Eulália Vinadé Chagas, Hilda Maria Rodrigues Moleda Constant, Vanessa Cristina Jacovas, Jacqueline Castro da Rocha, Carina Galves Crivella Steimetz, Maria Cristina Cotta Matte, Taís de Campos Moreira, Felipe Cezar Cabral

**Affiliations:** Brazilian Unified Health System Institutional Development Program (PROADI-SUS), Hospital Moinhos de Vento (HMV), Porto Alegre, RS, Brazil; University of Oklahoma Health Sciences Center, UNITED STATES

## Abstract

The use of telemedicine in ICUs has grown and is becoming increasingly recognized. However, its impact on PICUs remains unclear. This systematic review and meta-analysis aimed to evaluate whether telemedicine in the PICU has the potential to improve clinical and non-clinical outcomes. PubMed, Scopus, LILACS, and CINAHL electronic databases were searched to identify studies that assessed the impact of telemedicine on clinical outcomes, with no publication date restrictions. The reference lists of the selected articles were hand-searched for additional studies that had not been identified by the initial electronic search. Studies were included if they had a cohort design, used telemedicine, were conducted in PICUs or specialized PICUs, and were published in Portuguese, English, or Spanish. Two groups of reviewers independently screened titles and abstracts for inclusion. The same group of reviewers independently assessed the full-text articles for eligibility and extracted the following information: telecommunication method, intervention characteristics, patient characteristics, sample size, and main results. Studies were meta-analyzed using a random-effects model to estimate the pooled prevalence of PICU mortality and length of PICU stay. Risk of bias was assessed using the Newcastle-Ottawa Scale. Of 2703 studies initially identified, 2226 had their titles and abstracts screened. Of these, 53 were selected for full-text reading, of which 10 were included and analyzed. The main results of interest were length of PICU stay, number of deaths or mortality rate, and satisfaction of health professionals and family members. The results of meta-analysis show that the mortality rate reduced by 34% with an increase of the length of PICU stay in the PICUs with the use of telemedicine. Family members and health professionals were satisfied with the use of telemedicine. Telemedicine has the potential to improve PICU outcomes, such as mortality rate and family and staff satisfaction. However, it extended length of PICU stay in the studies included in this systematic review.

## Introduction

Telemedicine has become an option to overcome access barriers to health services due to its ability to overcome geographical barriers and to bridge health care gaps. The possibility of remotely providing specialty care services to patients living in underserved regions seems to contribute to positive outcomes in populations that would otherwise not have such access [1, 2]. In addition, telemedicine can also be used to support improvements in health indicators in units that need assistance in making diagnostic, therapeutic, and transport decisions, thus improving the efficiency of care [1, 3]. This type of health strategy has contributed to the discussion of complex cases and assisted in the lack of specialists in several areas, including the intensive care unit (ICU) [4, 5].

The use of telemedicine in ICUs has grown in recent years and is becoming increasingly recognized. In 2015, 13% of critical care services provided in the United States were supported by telemedicine [6]. Despite the lack of robustness, studies conducted in adult, pediatric, and neonatal ICUs have demonstrated several benefits of the implementation of telemedicine, such as lower mortality rates, shorter hospital stays [7], improved quality of daily care delivery practices, making them more specialized, greater adherence to evidence-based practices, lower complication rates, and improved patient and family satisfaction [6, 8]. In 2020 the use of telemedicine had an exponential increase in the world in reason of SARS-CoV-2 pandemic [9] becoming a necessary and important service to deliver a quality care to patients in despite of challenges, such as upfront investments, operational costs, and staff acceptance, which involves the culture and attitudes of medical and nursing teams [10].

Despite many studies involving the practice of telemedicine in ICUs, its impact on pediatric ICUs (PICUs) remains unclear; also, difficulties remain in comparing face-to-face and telemedicine interventions. In this context, understanding the impact of telemedicine on clinical practice is essential for the development of health strategies and new health policies. Therefore, the objective of this systematic review and meta-analysis was to evaluate whether telemedicine in the PICU has the potential to improve care outcomes and family and health professional´s satisfaction.

## Methods

### Protocol and registration

This systematic review and meta-analysis was conducted following the Preferred Reporting Items for Systematic Reviews and Meta-Analyses (PRISMA) guidelines [11], and its protocol was registered with the International Prospective Register of Systematic Reviews (PROSPERO), under registration number CRD42020148180.

### Data sources and search strategy

The keywords required for our searches were identified using the participants, interventions, comparators, outcomes, and study design (PICOS) approach [12, 13] in order to guide the search and obtain the specific studies that would best serve our review. A search strategy was developed for each database using specific search terms (S1 File). We performed the search of the following databases through April 16, 2021: MEDLINE (via PubMed), Scopus, Latina American and Caribbean Health Sciences Literature (LILACS), and Cumulative Index to Nursing and Allied Health Literature (CINAHL) electronic databases. The reference lists of the selected articles were hand-searched for additional studies that had not been identified by the initial electronic search. The literature search was performed under the guidance of a librarian with experience in database searches.

### Eligibility criteria

Prospective and retrospective cohort studies of PICUs published in Portuguese, English, or Spanish were included.

#### Study design

Only concurrent and non-concurrent cohort studies were included. Material from the gray literature, descriptive studies, and case reports were excluded.

#### Participants and definitions

Only studies with a sample of pediatric patients admitted to a general PICU, cardiac PICU, or adult ICU with beds for pediatric patients were eligible for inclusion. The latter ICU type was included because less severe patients were allocated to this adult ICU in a rural hospital, and remote care was provided by telemedicine by pediatric intensivists. No sociodemographic, socioeconomic, or disease severity restrictions were imposed. Studies using synchronous and asynchronous telemedicine were included.

#### Outcomes

Studies reporting participants’ clinical outcomes, such as mortality rate or number of deaths and length of PICU stay, and non-clinical outcomes, such as satisfaction with the use of telemedicine and quality of care, were assessed. Mortality rate was calculated by dividing the number of deaths by the number of patients discharged, as reported in the selected studies [14].

### Data collection and validity assessment

#### Study selection

Two groups of reviewers (MC/VJ and JC/HC) independently screened titles and abstracts identified by the initial search. Abstracts that did not provide sufficient information on the eligibility criteria were kept for full-text reading. The same group of reviewers independently assessed the full-text articles for eligibility. Disagreements were resolved by consensus in meetings or by consulting a third reviewer (TM) for arbitration. In this systematic review and meta-analysis telephone calls and videocalls among physicians or among physician and others health professionals was considered as telemedicine interventions.

#### Data extraction

Two groups of reviewers (MC/VJ and JC/HC) extracted the following information from the selected studies: authors, year of publication, country where the study was conducted, objective, sample size and characteristics, telemedicine model used, follow-up period, and clinical and non-clinical outcomes.

#### Risk of bias

The quality of the included studies was assessed by the Newcastle-Ottawa Scale (NOS) [15]. The NOS assesses studies in 3 categories: selection, comparability of the groups (cohorts), and outcome (cohorts). A maximum of 9 stars is awarded for study quality, rated as high (7 to 9 stars), moderate (4 to 6 stars), or low (1 to 3 stars) [15]. It is a widely used instrument despite some limitations, as mentioned in the study by Luchini *et al*. [16]. The NOS classification of the included studies is available in S1 Table.

### Data analysis

Initially, we performed a descriptive analysis of the studies, including patient characteristics, telemedicine model, follow-up duration, outcomes, and pre- and post-telemedicine results (Tables 1 and 2). Publication bias across studies wasn’t evaluated using graphical or statistical methods because we have less than ten studies included in the meta-analysis, which implies in a low power of the test [17].

**Table 1:** Study characteristics.

**Title and Reference**	**Cohort collection year**	**Country**	**Study design**	**Sample size**	**Telemedicine model**	**Objective**
Creation of a rudimentary electronic pediatric intensive care unit model to explore resident-attending communication [18]	2016–2017	USA	Prospective cohort	40	Synchronous	To determine if using telemedicine communication between in-house pediatric residents and at-home attending intensivists impacts the rate of attending return to the hospital and improves resident education.
Impact of telemedicine on severity of illness and outcomes among children transferred from referring emergency departments to a children’s hospital PICU [19]	2010–2014	USA	Retrospective cohort	1106	Synchronous	To compare the severity of illness and outcomes among children admitted to a children’s hospital PICU from referring emergency departments with and without access to a pediatric critical care telemedicine program.
Patient outcomes of an international telepediatric cardiac critical care program [20]	2011–2012	Colombia and USA	Retrospective cohort	378	Synchronous	To describe variables associated with patient outcome during the implementation of an international pediatric cardiac critical care telemedicine program.
Telemedicine in pediatric critical care: a retrospective study in an international extracorporeal membrane oxygenation program [21]	2010–2015	Colombia and USA	Retrospective cohort	106	Asynchronous and synchronous	To evaluate whether telemedicine in the cardiac ICU can be used to enhance the delivery of quality interventions.
A more rapid, rapid response [22]	2014	USA	Prospective cohort	91	Synchronous	To evaluate whether a FaceTime video call between the staff at the bedside and the critical care physician will allow the implementation of potentially life-saving therapies earlier than the current average response (4.5 min).
The implementation of a synchronous telemedicine platform linking off-site pediatric intensivists and on-site fellows in a pediatric intensive care unit: A feasibility study [23]	2017–2018	Canada	Prospective cohort	14	Synchronous	To assess the feasibility of implementing a synchronous telemedicine platform in a pediatric intensive care unit (STEP-PICU).
Use of telemedicine to provide pediatric critical care inpatient consultations to underserved rural Northern California [24]	1997/1998 and 2000/2002	USA	Retrospective and prospective cohort	163	Synchronous	To report a novel application of telemedicine and to assess the resulting quality and satisfaction of care.
Telemedicine in pediatric cardiac critical care [25]	2010	Colombia and USA	Prospective cohort	53	Synchronous	To describe international telemedicine experience in pediatric cardiac critical care.
The Effect of Telemedicine on Resource Utilization and Hospital Disposition in Critically Ill Pediatric Transport Patients [26]	2012–2015	USA	Retrospective cohort	212	Synchronous	To demonstrate that the use of telemedicine improves the patient’s understanding of the disease process compared with verbal description by phone.
The use of telemedicine to provide pediatric critical care consultations to pediatric trauma patients admitted to a remote trauma intensive care unit: a preliminary report [27]	2000–2002	USA	Retrospective and prospective cohort	97	Synchronous	To describe a pilot telemedicine project that allows an adult ICU to obtain nontrauma, nonsurgical-related pediatric critical care consultations for acutely injured children.

**Table 2:** Study characteristics: Outcomes and results.

Title and Reference	Follow-up duration (months)	Outcome measures	Results pre-telemedicine/no telemedicine	Results post-telemedicine	Study outcome
Creation of a rudimentary electronic pediatric intensive care unit model to explore resident-attending communication [18]	8	a) Length of stay; b) Clinical severity; c) Ease of use by intensivists; d) Resident comfort.	a) Length of stay: 1.5 days; b) Clinical severity: mean, 3.7; c) Ease of use: mean, 10; d) Comfort: mean, 10.	a) Length of stay: 1 day; b) Clinical severity: mean, 4.5; c) Ease of use: mean, 10; d) 4) Comfort: mean, 10.	There was no significant difference between pre- and post-telemedicine.
Impact of telemedicine on severity of illness and outcomes among children transferred from referring emergency departments to a children’s hospital PICU [19]	48	a) Length of stay; b) Clinical severity; c) Number of deaths; d) Time of admission; e) Day of admission; f) Disposition; g) Transport distance.	a) Length of stay: mean, 3.8 days; b) Clinical severity: more ill (PRISM III score = 4); c) Number of deaths: 23 patients; d) Time of admission: nighttime admission = 335 patients; e) Day of admission: weekend admission = 153 patients; f) Disposition: general care floor, n = 297; home, n = 150; step-down unit, n = 15; another ICU, n = 8; other, n = 54; g) Transport distance: 63.1 miles.	a) Length of stay: mean, 3.1 days; b) Clinical severity: less ill (PRISM III score = 3.2); c) Number of deaths: 14; d) Time of admission: nighttime admission = 326; e) Day of admission: weekend admission = 185; f) Disposition: general care floor, n = 334; home, n = 184; step-down unit, n = 13; another ICU, n = 9; other, n = 42; g) Transport distance: 72.4 miles	There were significant differences in severity, time of admission, and distance between pre- and post-telemedicine. The use of telemedicine resulted in fewer nighttime admissions, lower illness severity, shorter length of stay, and lower mortality, although the latter two have no significant difference.
Patient outcomes of an international telepediatric cardiac critical care program [20]	10	a) Length of stay; b) Number of deaths.	a) Length of stay: mean, 11 days; b) Number of deaths: 66 patients;	a) Length of stay: mean, 17 days; b) Number of deaths: 13 patients.	There were no significant differences in mortality. During the use of telemedicine in surgical patients, preoperative length of stay was significantly shorter.
Telemedicine in pediatric critical care: a retrospective study in an international extracorporeal membrane oxygenation program [21]	48	a) Length of stay; b) Survival.	a) Length of stay: mean, 20 days; b) Survival: 17 patients.	a) Length of stay: mean, 41 days; b) Survival: 59 patients.	There was no significant difference between pre- and post-telemedicine in the outcomes assessed.
A more rapid, rapid response [22]	07	a) Response time; b) Number of interventions performed by physicians; c) Satisfaction (staff).	a) Time to arrive at bedside: mean, 3.7 min; b) Number of interventions: mean, 1.9; c) NA.	a) Time to establish FaceTime interface: mean, 2.6 min; b) Number of interventions: mean, 1.4; c) Satisfaction (staff): 20% felt that the iPad was difficult to use / 67% felt that the iPad was beneficial to the patient’s care / 50% stated that the iPad reduced their anxiety during the rapid response / 6% stated that they were not confident in the recommendations received via the IPad.	There was a significant difference in response time, which was faster when telemedicine was used.
The implementation of a synchronous telemedicine platform linking off-site pediatric intensivists and on-site fellows in a pediatric intensive care unit: a feasibility study [23]	06	a) System quality; b) Quality of technical support; c) Data quality; d) Overall satisfaction; 5) System benefits; e) Use of the new system.	NA	a) System quality: 4.40/10; b) Data quality: 5.52/10; c) Quality of technical support: 5.81/10; d) System use: 2.39/10; e) Overall satisfaction: 3.53/10; f) System benefits: 2.93/10.	There were no comparisons.
Use of telemedicine to provide pediatric critical care inpatient consultations to underserved rural Northern California [24]	24	a) Number of deaths; b) Efficiency of care in the PICU; c) Family and staff satisfaction.	a) Number of deaths: 3 patients; b) Efficiency of care in the PICU: 29.6%; c) Family and staff satisfaction: NA	a) Number of deaths: 1 patient; b) *Efficiency of care in the PICU: 53.9%; c) Satisfaction: family, 4.05/5; staff, 4.54/5.	There were no comparisons in the cohorts under study.
Telemedicine in pediatric cardiac critical care [25]	10	a) Staff satisfaction.	NA	a) Staff satisfaction: Very satisfied, 6/8.	All staff members agreed that the telemedicine consultation system was very useful. There were no statistical comparisons.
The effect of telemedicine on resource utilization and hospital disposition in critically Ill pediatric transport patients [26]	24	a) Disposition; b) Code blue (protocol for medical emergency in cardiac arrest patients).	a) Disposition: PICU, 10.6%; b) Code blue: 3;	a) 1) Disposition: PICU, 34.4%; 2) Code blue: 3.	There was a significant difference in disposition patterns, since telemedicine allowed better assessment of patients.
The use of telemedicine to provide pediatric critical care consultations to pediatric trauma patients admitted to a remote trauma intensive care unit: a preliminary report [27]	24	a) Length of stay; b) Number of deaths; c) Parental satisfaction; d) Staff satisfaction.	a) Length of stay: 3.5 days; b) Number of deaths: 6 patients; c) NA; d) NA.	a) Length of stay: 5.9 days; b) Number of deaths: 0; c) Parental satisfaction: 3.8/5; d) Staff satisfaction: 4.75/5.	There was no comparison for length of stay or mortality. There was no significant difference in satisfaction.

### Meta-analysis

The meta-analysis was performed using data on length of PICU stay and mortality. We pooled the prevalence estimates from the included studies using a random-effects model, with the DerSimonian-Laird estimator for between-study variance. The results were presented as mean difference (MD) for length of stay (in days) or as measures of relative risk (RR) for mortality. In the absence of standard deviation (SD) values for a specific study, we used the arithmetic mean of the SDs of the other studies in which the data were available. This method is preferable because it offers more stable results than just omitting studies with missing summary of variance statistics [28].

The statistical heterogeneity of treatment effects between studies was assessed using the Cochran Q test and the I^2^ inconsistency test [29]. Where possible, subgroup analyses were performed to investigate the main sources of heterogeneity between studies, such as follow-up duration. The studies included were divided into those with a follow-up 24 months and with more than 24 months because there could be a care bias to the patient the longer the follow-up time.

After data extraction, if the results could not be summarized by meta-analysis, as satisfaction, descriptive synthesis was performed to present the findings. In some studies, the satisfaction was about health professional’s satisfaction [22–25, 27] and in others it was about family satisfaction [24, 27] and the results were in different scales. The satisfaction results were heterogeneous, which as summarized by meta-analysis, could not be taken in consideration.

All analyses were performed using R, version 3.0.2 (R-Project, 2014, Los Angeles, CA, USA), using the meta and metafor packages [30]. *P*-values <0.05 were considered statistically significant.

## Results

### Study selection

**Fig 1:**
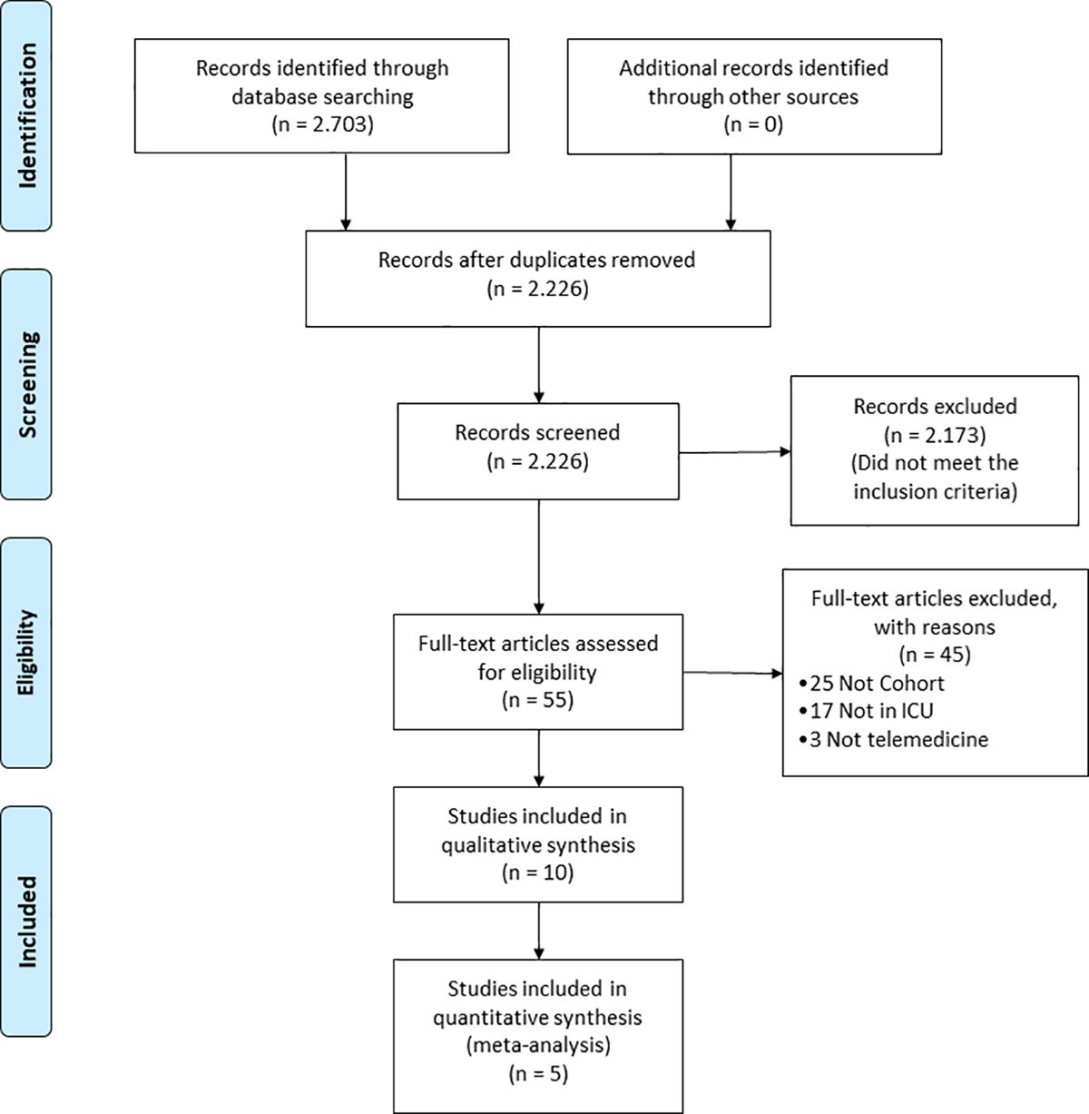
Prisma flow diagram. Flow diagram illustrating Search strategy.

Of 2703 records identified through the search strategy, 2226 had their titles and abstracts screened. Of these, 55 were selected for full-text reading, of which 10 [18–27] were included in the present study (Fig 1).

### Study characteristics

The sample size of the 10 included studies [18–27] ranged from 14 [22] to 582 [18] patients. The duration of cohort follow-up ranged from 6 [22] to 48 [18, 20] months. Five studies were conducted in general PICUs [18, 19, 22, 23, 26], 3 in cardiac PICUs [19, 20, 24], and 2 in adult ICUs with beds for pediatric patients [24, 27]. Length of PICU stay ranged from 1 to 41 days. Mortality rate ranged from 0 [27] to 45.9% [21] for cohorts with telemedicine and from 2.6 [24] to 70.1% [21] for cohorts without telemedicine. Some studies have a high mortality rate [19–21] in reason of the patient´s severity illness in transport to a PICU or because they were cardiac patients in a CICU. These rates were included in the meta-analysis of mortality [19–21, 24, 27]. Even the lower number of studies included in the meta-analysis, we follow the guidelines of Viechtbauer et al., indicating that the effects do not depend on a single study, therefore to exclude the outliers or influential cases may be an error, once small or large effects could be a result of chance alone [31]. Based on the analysis of satisfaction, both family members and health professionals were satisfied with the use of telemedicine in the PICU (Tables 1 and 2).

### Synthesis of results

Of the 10 cohort studies included in this systematic review, 5 assessed the number of deaths in the PICU [19–21, 24, 27] and 5 assessed patients’ length of PICU stay [18–21, 27].

**Fig 2:**
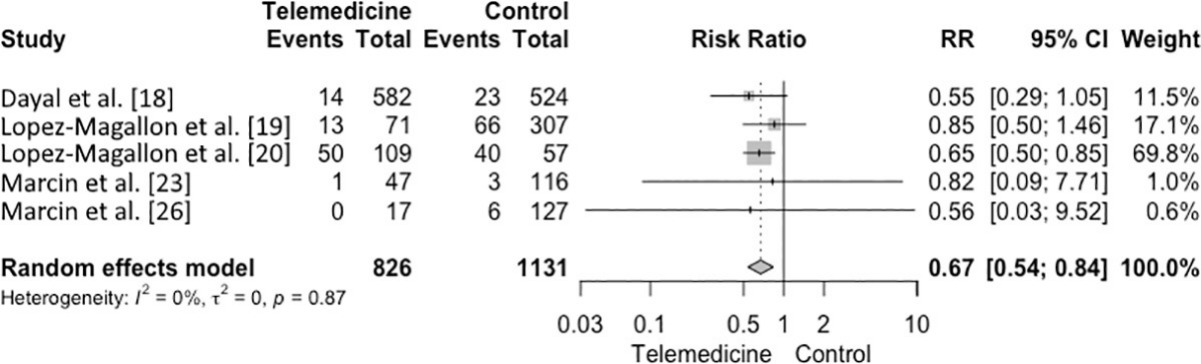
Forest plot. Mortality rate.

The mortality rate decreased by 34% in the PICUs that implemented the use of telemedicine, ranging from 16 to 46% (RR 0.67, 95% CI 0.54–0.84, I^2^ = 0%; *p* = 0.87) (Fig 2). This result shows that PICUs without the support of telemedicine tend to be a risk factor for mortality.

**Fig 3:**
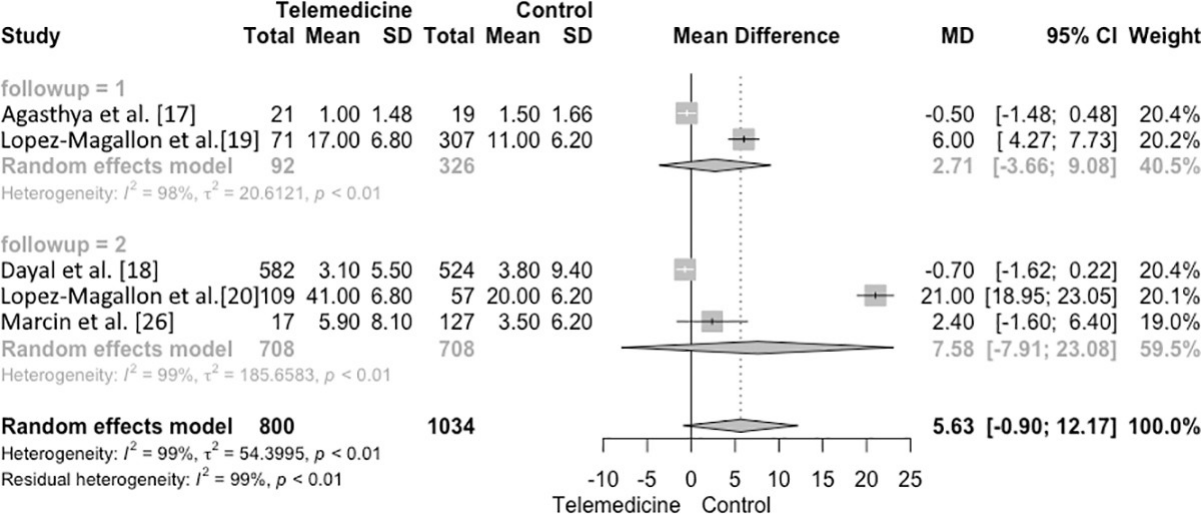
Forest plot. Length of PICU stay.

The mean length of PICU stay was 5.63 days (95% CI −0.9 to 12.17, I2 = 99%; p < 0.01) (Fig 3). Subgroup analyses were performed to explore heterogeneity among studies. When studies with a follow-up of 24 months or less [18, 20] were compared to those with more than 24 months of follow-up [19, 21, 27], length of PICU stay was MD = 2.71 (95% CI −3.66 to 9.08; I2 = 98%) in the group ≤24 months vs MD = 7.58 (95% CI −7.91 to 23.08, I2 = 99%) in the group >24 months.

In addition, when evaluating methodological differences between studies, 2 studies [20, 21] were conducted in cardiac PICUs, where patients tend to have higher severity of illness than those in general PICUs [32]. Although the lengths of stay were 17 days and 41 days, respectively, we decided to keep these studies in the analyses in order not to reduce the sample size of the present study. The studies were carefully performed in a subgroup´s analyses and as in the mortality´s meta-analysis we follow the recommendation of Viechtbauer et al. [31].

### Risk of bias within studies

All cohorts included in the present review were assessed by the NOS [15]. Study quality was rated as low in 1 study [22], moderate in 3 [22, 25, 27], and high in 6 [18–21, 24, 26].

## Discussion

Telemedicine has the potential to improve PICU outcomes. Our results demonstrated a 34% reduction in mortality in the PICUs that implemented the use of telemedicine in their clinical practice, suggesting that specialized medical assistance, even at a distance, can optimize and improve care for critically ill patients, reducing the incidence of complications associated with high mortality rates [33]. Adult ICUs that used telemedicine also showed an improvement in clinical outcomes, evidenced by a reduction in mortality rates, a decrease in interhospital transfers, and an improvement in clinical practices in these units [34]. The use of telemedicine allows specialized care expansion, creating opportunities to others health professional’s delivery care to the patients with an adequate flow of information and experience, which qualifies the treatment, medical management and makes the difference in the mortality rate. Telemedicine is a tool which makes easier the communication between health professionals 24/7 in the decision-making resulting the reduction in mortality rate [19].

Regarding length of PICU stay, the results of the meta-analysis showed a difference between PICUs with and without the use of telemedicine, with an increase of 5.63 days in length of stay in units using telemedicine [18–21, 27]. One possible explanation is that shared care between teams and the provision of specialty care services by specialists reduced potential complications, increased patient survival, and, consequently, extended length of PICU stay [32].

Of the 10 included studies, only 5 [22–25, 27] evaluated PICU staff satisfaction with the use of telemedicine in patient care, while 2 studies [24, 27] also evaluated family satisfaction. In general, family members were satisfied with the use of telemedicine in the PICU. Satisfaction was mainly related to efficient communication, high quality of care, and audiovisual quality [24, 27]. Family members who are able to be more often at their child’s bedside and to remotely participate in PICU rounds tend to be more satisfied, less stressed and feel more involved in the daily care of their child [35]. Health care teams were also satisfied with the use of telemedicine, since the technology employed proved to be easy to use, contributed to improving the care provided to the patient, and reduced their level of anxiety [22]. Kleinpell et al. [36], in a study of nurses’ perceptions of intensive care telemedicine, highlighted that using telemedicine, on a daily basis, improves patient care, increases productivity, and makes the nurse’s job easier, as it is performed in collaboration with other health professionals at a distance. Kissi et al. [37] reported that telemedicine supports physicians’ responsibilities related to service delivery and general medical decisions. However, to produce high levels of satisfaction among physicians, telemedicine must integrate physicians’ and their patients’ needs, have high audiovisual quality, and be easy to use.

## Limitations

Despite the suggested improvement in outcomes in PICUs using telemedicine, this systematic review demonstrated that there is a lack of robust evidence in the current literature [38]. There are so many forms of this approach and, as it turns out, quite a breadth is included in this analysis. The settings vary drastically, including pediatric floors, distant ERs and outlying hospitals without subspecialists. However, they were carefully evaluated for inclusion in the study. Several studies allow only limited generalizations due to methodological problems [25], sample selection issues [27], inadequate follow-up [23], and unsuitable comparisons between cohorts [20]. Another limitation of the present study is the inclusion of PICUs with different clinical profiles (a strategy adopted due to the reduced number of studies available in the literature), which may introduce bias in the results. Regarding satisfaction with the use of telemedicine, it was difficult to evaluate the results together given the lack of homogeneity in satisfaction assessments across the studies, as the use of different scales. Regarding outcomes, only part of the studies were used to assess each endpoint because of missing information. Satisfaction assessment is essential in telemedicine, although there is currently no validated tool to measure satisfaction in the PICU [39]. In addition, because our search was limited to articles published in English, Portuguese, or Spanish, it is possible that other relevant studies may have not been detected.

## Conclusions

The use of telemedicine in PICUs has grown recently, with a positive impact on clinical outcomes, particularly a significant reduction in mortality, in addition to contributing to the increased satisfaction of family members and health professionals with patient care. However, a longer length of stay was observed in these units. We suggest that further well-designed studies be performed to better understand the impact and real benefits of using telemedicine in clinical practice.

## Supporting information

S1 Checklist PRISMA 2009 checklist. (DOC)

S1 File Search strategy for PubMed, Scopus, LILACS, and CINAHL. (DOCX)

S1 Table Classification of included studies according to the Newcastle-Ottawa Scale (NOS). (DOCX)

S2 Table Individual components of the risk of bias assessment. (XLSX)
