# Supplementary material for: The use of telemedicine in the PICU: A systematic review and meta-analysis
Source: PLoS One. 2021 May 28;16(5):e0252409. doi: 10.1371/journal.pone.0252409 (PMC8162650; doi:10.1371/journal.pone.0252409)
Supplement: S1 File — (DOCX) [file pone.0252409.s002.docx]

**Supporting Information 1 file**. Search strategy for PubMed, Scopus, LILACS, and CINAHL.

1. PubMed:
2. Pediátrico: (Pediatrics[mh:NoExp] OR Child[mh] OR Infant[mh] OR Adolescent[mh] OR child[tw] OR children[tw] OR childhood[tw] OR newborn*[tw] OR adolescen*[tw] OR paediatric*[tw] OR pediatric*[tw] OR teen*[tw] OR Youth*[tw]);
3. Cuidado crítico: (Critical Care[mh] OR Intensive Care Units, Pediatric[mh] OR Critical care[tw] OR Intensive care[tw]);
4. Telemedicina: (Telemedicine[mh] OR Telemedicine[tw] OR Tele medicine[tw] OR Tele-medicine[tw] OR Telehealth[tw] OR Tele health[tw] OR Tele-health[tw] OR eHealth[tw] OR Mobile Health[tw] OR Remote health[tw] OR Virtual health[tw] OR Mobile medicine[tw] OR Remote medicine[tw] OR Virtual medicine[tw] OR Telerehabilitation[tw] OR Tele-rehabilitation[tw] OR Tele rehabilitation[tw] OR Telemonitoring[tw] OR Tele-ICU[tw] OR Tele ICU[tw] OR Remote monitoring[tw] OR Remote-monitoring[tw]);
5. **1 AND 2 AND 3.**
6. Scopus:
7. Pediátrico: INDEXTERMS(newborn OR infant OR baby OR toddler OR "school child" OR child OR juvenile OR adolescent) OR TITLE-ABS-KEY(child OR children OR childhood OR newborn* OR adolescen* OR paediatric* OR pediatric* OR teen* OR Youth*);
8. Cuidado crítico: INDEXTERMS("intensive care" OR "intensive care unit") OR TITLE-ABS-KEY("Critical care" OR "Intensive care");
9. Telemedicina: INDEXTERMS(telemedicine) OR TITLE-ABS-KEY(Telemedicine OR "Tele medicine" OR "Tele-medicine" OR Telehealth OR "Tele health" OR "Tele-health" OR eHealth OR "Mobile Health" OR "Remote health" OR "Virtual health" OR "Mobile medicine" OR "Remote medicine" OR "Virtual medicine" OR Telerehabilitation OR "Tele-rehabilitation" OR "Tele rehabilitation" OR "Tele-ICU" OR "Tele ICU");
10. **1 AND 2 AND 3.**
11. Lilacs:
12. Pediátrico: (mh:Pediatrics OR mh:M01.060.406* OR mh:M01.060.703* OR mh:M01.060.057* OR tw:child* OR tw:crianca* OR tw:nino OR tw:ninos OR tw:nina OR tw:ninas OR tw:childhood OR tw:infancia OR tw:newborn* OR tw:"recém nascid*" OR tw:"recien nacid*" OR tw:adolescen* OR tw:paediatric* OR tw:pediatric* OR tw:teen* OR tw:joven* OR tw:juventud OR tw:jovem OR tw:jovens OR tw:juventude);
13. Cuidado crítico: (mh:N02.421.585.190* OR mh:N02.278.388.493.390* OR tw:"Critical care" OR tw:"cuidados criticos" OR tw:"cuidado critico" OR tw:"cuidados intensivos" OR tw:"cuidado intensivo");
14. Telemedicina: (tw:Telemedicina OR tw:eSalud OR tw:"Ciber Salud" OR tw:"Ciber-Salud" OR tw:Cibersalud OR tw:"Medicina 2.0" OR tw:"Salud 2.0" OR tw:"Salud Conectada" OR tw:"Salud Digital" OR tw:"Salud Electronica" OR tw:"Salud Mueble" OR tw:"Salud Omnipresente" OR tw:"Salud Pervasiva" OR tw:"Salud Ubicua" OR tw:Telesalud OR tw:Teleasistencia OR tw:Telecuidado OR tw:Telecura OR tw:"Teleservicios Sanitarios" OR tw:"Teleservicios de Salud" OR tw:mSalud OR tw:uSalud OR tw:"Ciber Saude" OR tw:"Ciber-Saude" OR tw:Cibersaude OR tw:"Medicina 2.0" OR tw:"Saude 2.0" OR tw:"Saude Conectada" OR tw:"Saude Digital" OR tw:"Saude Eletrônica" OR tw:"Saude Móvel" OR tw:"Saude Onipresente" OR tw:"Saude Pervasiva" OR tw:"Saude Ubiqua" OR tw:Telessaude OR tw:"e-Saude" OR tw:eSaude OR tw:"Tele-Serviços em Saude" OR tw:Teleassistencia OR tw:"Telesservicos de Saude" OR tw:"Telesservicos em Saude" OR tw:"Telesservicos na Saude" OR tw:mSaude OR tw:uSaude);
15. **1 AND 2 AND 3.**
16. CINAHL:
17. Pediátrico: (MH Pediatrics OR MH Child OR MH Infant OR MH Adolescent OR TX child OR TX children OR TX childhood OR TX newborn* OR TX adolescen* OR TX paediatric* OR TX pediatric* OR TX teen* OR TX Youth*);
18. Cuidado crítico: (MH "Critical Care" OR MH "Intensive Care Units, Pediatric" OR TX "Critical care" OR TX "Intensive care");
19. Telemedicina: (MH Telemedicine OR TX Telemedicine OR TX "Tele medicine" OR TX "Tele-medicine" OR TX Telehealth OR TX "Tele health" OR TX "Tele-health" OR TX eHealth OR TX "Mobile Health" OR TX "Remote health" OR TX "Virtual health" OR TX "Mobile medicine" OR TX "Remote medicine" OR TX "Virtual medicine" OR TX Telerehabilitation OR TX "Tele-rehabilitation" OR TX "Tele rehabilitation" OR TX "Tele-ICU" OR TX "Tele ICU");
20. **1 AND 2 AND 3.**
