# Supplementary material for: The use of telemedicine in the PICU: A systematic review and meta-analysis
Source: PLoS One. 2021 May 28;16(5):e0252409. doi: 10.1371/journal.pone.0252409 (PMC8162650; doi:10.1371/journal.pone.0252409)
Supplement: S1 Table — (DOCX) [file pone.0252409.s003.docx]

**S1 Table**. Classification of included studies according to the Newcastle-Ottawa Scale (NOS) [13]:

| **Title and Reference** | **Selection** | **Comparability** | **Outcome** | **Classification** |
| --- | --- | --- | --- | --- |
| Creation of a rudimentary  electronic pediatric intensive care unit model to explore resident-attending communication [15] |  |  |  | High |
| Impact of telemedicine on severity of illness and outcomes among children transferred from referring emergency departments to a Children’s Hospital PICU [16] |  |  |  | High |
| Patient outcomes of an international telepediatric cardiac critical care program [17] |  |  |  | High |
| Telemedicine in pediatric critical care: a retrospective study in an international extracorporeal membrane oxygenation program [18] |  |  |  | High |
| A more rapid, rapid response [19] |  |  |  | Moderate |
| The implementation of a synchronous telemedicine platform linking off-site pediatric intensivists and on-site fellows in a  pediatric intensive care unit: A feasibility study [20] |  |  |  | Low |
| Use of telemedicine to provide pediatric critical care inpatient consultations to underserved rural Northern California [21] |  |  |  | High |
| Telemedicine in pediatric cardiac critical care [22] |  |  |  | Moderate |
| The effect of telemedicine on resource utilization and hospital disposition in critically Ill pediatric transport patients [23] |  |  |  | High |
| The use of telemedicine to provide pediatric critical care consultations to pediatric trauma patients admitted to a remote trauma intensive care unit: A preliminary report [24] |  |  |  | Moderate |

The NOS assessed the studies in 3 categories: selection, comparability of the groups (cohorts), and outcome (cohorts). Classification resulted from the sum of the scores obtained in each category, as follows: 7 to 9 stars=high quality, 4 to 6 stars=moderate quality, and 1 to 3 stars=low quality.
